# Draft Genome Sequence of *Paenibacillus etheri* sp. nov. SH7^T^, a Methyl Tert-Butyl Ether Degrader

**DOI:** 10.1128/genomeA.01696-15

**Published:** 2016-02-18

**Authors:** Jessica Purswani, Isabel M. Guisado, Jesus Gonzalez-Lopez, Clementina Pozo

**Affiliations:** a Environmental Microbiology Group, Institute of Water Research, University of Granada, Granada, Spain; b Department of Microbiology, University of Granada, Granada, Spain

## Abstract

We report here the draft genome sequence of *Paenibacillus etheri* sp. nov. SH7^T^ (= CECT 8558^T^ = DSM 29760^T^), isolated from a hydrocarbon-contaminated soil pilot plant in Granada, Spain. The bacterium was isolated and sequenced due to its methyl tert-butyl ether (MTBE)-degrading properties.

## GENOME ANNOUNCEMENT

The genus *Paenibacillus* encompasses approximately 200 species to date (available in the GenBank database), with a diversity of niches, including soil, freshwater, human, and plant rhizosphere habitats. *Paenibacillus etheri* sp. nov. SH7^T^ (1) was isolated from a hydrocarbon-contaminated soil pilot plant in Spain (2, 3), using enrichment techniques with methyl tert-butyl ether (MTBE) (4), a gasoline additive and common groundwater pollutant. *P. etheri* has great potential for being used in MTBE bioremediation technologies, due to its MTBE-degrading capabilities (80 mg/liter MTBE in 7 days) (2). There are very few MTBE-degrading microorganisms with published bacterial genomes; hence, a greater scope of unique or diverse metabolic pathways can be found by sequencing MTBE-tolerant/-degrading microorganisms.

Whole-genome sequencing of *P. etheri* SH7^T^ was performed using short-read (approximately 200- to 300-bp) and long-read (approximately 1-kbp) libraries with the Nextera XT kit (Illumina), and sequenced using 150-bp paired-end reads in the MiSeq Illumina platform. For the long-read library, an initial DNA quantity of 20 ng was used (instead of the default short-read library protocol of 1 ng). A total of 3.1 million reads were generated and reached a genome coverage of >50-fold. Genome assembly was performed with Velvet (k-mer, 99), resulting in 112 contigs, with an *N*_50_ of 534,899 bp and genome size of 6,634,505 bp. The sequences were ordered using genome alignment software (Mauve 2.3.1), using *Paenibacillus* sp. Y412MC10 as a reference. Four contigs of <200 bp were removed.

The resulting assembled and ordered sequences were annotated by the NCBI Prokaryotic Annotation Pipeline, generating a genome size of 6,633,655 bp, with a mean G+C content of 43.1%. NCBI annotation found 5,640 coding sequences (CDSs) and 136 RNA molecules. A Blast comparison of the SH7^T^ 16S RNA gene (accession no. KC625558) with type strains returned the following % identities for *Paenibacillus* species (query coverage of 100%, unless stated): *P. odorifer*, 98%; *P. borealis*, 97%; *P. typhae*, 97% (query coverage, 97%); *P. wynnii*, 96%; and *P. graminis*, 95%. A genome comparison of *P. etheri* SH7^T^ revealed that its genome size is within the range of its genus; however, the G+C content is lower, as *P. graminis* RSA19 (accession no. PRJNA202950), *Paenibacillus* sp. Y412MC10 (accession no. NC_013406), and *P. polymyxa* (accession no. NC_014483.1) have genome sizes of 6.9, 7.1, and 5.4Mbp and G+C contents of 50.3, 51.2, and 45.8%, respectively.

### Nucleotide sequence accession numbers.

This whole-genome shotgun project has been deposited at DDBJ/EMBL/GenBank under the accession no. LCZJ00000000. The version described in this paper is the second version, LCZJ02000000.
